# Remote *Salmonella* Enteritidis Bacteremia and Subsequent Covered Stent Infection: Clinical and Mechanistic Insights from a Rare Vascular Case

**DOI:** 10.3390/jcm15145492

**Published:** 2026-07-13

**Authors:** Bartłomiej Antoń, Milena Michalska, Michał Macech, Witold Rongies, Sławomir Nazarewski, Zbigniew Gałązka

**Affiliations:** 1 Department of General, Vascular, Endocrine and Transplant Surgery, Medical University of Warsaw, ul. Banacha 1a, 02-097 Warsaw, Poland; 2 Department of Rehabilitation, Medical University of Warsaw, ul. Banacha 1a, 02-097 Warsaw, Poland

**Keywords:** popliteal artery pseudoaneurysm, covered stent infection, *Salmonella* Enteritidis, vascular graft infection, endovascular repair, autologous vein bypass, limb salvage

## Abstract

**Background**: Endovascular repair with covered stent is an established minimally invasive treatment for popliteal artery lesions, particularly in elderly or high-risk patients. Infectious complications are exceptionally rare but may result in arterial destruction, limb loss, and death. *Salmonella* species demonstrate a well-recognized affinity for diseased arterial walls and prosthetic vascular material. **Case Presentation**: An 82-year-old man with stage G4 chronic kidney disease and previous nephrectomy for renal cell carcinoma, complicated by *Salmonella Enteritidis* septic shock 20 years earlier, presented with acute left lower limb ischemia caused by a post-traumatic popliteal artery pseudoaneurysm. Urgent endovascular repair was performed using a 6 × 100 mm covered stent (Viabahn) with adjunctive angioplasty of the anterior tibial artery. Two weeks later, he was readmitted with fever, severe popliteal pain, local erythema, and elevated inflammatory markers. Imaging demonstrated early stent occlusion with a large peri-graft abscess and contained arterial rupture. Emergency open conversion included radical debridement, complete graft explantation, and popliteal-to-posterior tibial bypass using an autologous great saphenous vein. Cultures grew *Salmonella* Enteritidis. Retrospective history revealed a previously undocumented episode of *Salmonella* Enteritidis bacteremia approximately 20 years earlier. At 2-year follow-up, the patient remained free of recurrent infection with a patent vein graft and preserved ambulatory function. **Conclusions**: Early *Salmonella* Enteritidis infection of a popliteal covered stent is an exceptionally rare but life-threatening complication. This case suggests that remote *Salmonella* bacteremia may represent a potential, hypothesis-generating risk factor for prosthetic graft infection. Prompt graft explantation followed by radical debridement and autologous venous reconstruction remain essential for durable limb salvage.

## 1. Introduction

Popliteal artery aneurysms are the most common peripheral arterial aneurysms. Although frequently asymptomatic, they carry a substantial risk of thromboembolism, acute limb ischemia and, less often, rupture; timely surgical or endovascular treatment is therefore indicated once accepted thresholds are met [1]. Post-traumatic popliteal pseudoaneurysms are a distinct and far less common entity, but they share this thrombotic and ischemic risk and likewise require prompt repair.

Over the last two decades, covered stents have become an effective, minimally invasive alternative to open bypass, particularly in elderly or high-risk patients with significant comorbidity [2,3]. Although patency and limb-salvage outcomes are satisfactory in selected cohorts, every prosthetic endograft carries a life-long risk of device-related infection [4]; such infection after endovascular treatment of popliteal lesions nonetheless remains exceedingly rare [2,5].

Among vascular pathogens, *Salmonella* species merit particular attention because of their marked tropism for atherosclerotic arteries, aneurysmal walls and synthetic vascular material [4,6]. Once established, *Salmonella* vascular infection follows an aggressive course, with rapid tissue destruction, pseudoaneurysm formation, secondary rupture and high mortality when treatment is delayed [7]. Even so, *Salmonella* infection after endovascular peripheral intervention is exceptionally rarely reported [8].

**Figure 1:**
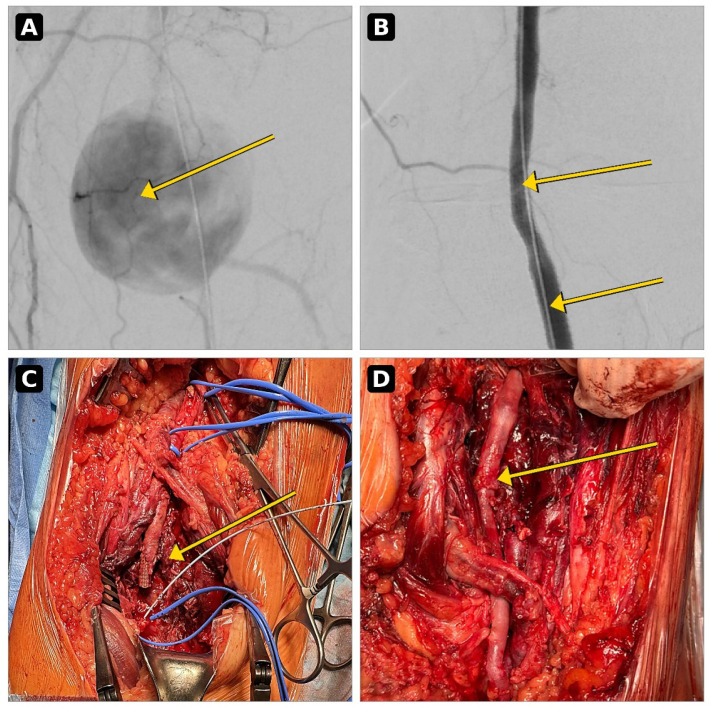
Chronological overview of the diagnostic (**A**), endovascular treatment (**B**), infectious/intraoperative (**C**), and reconstructive (**D**) phases of management. (**A**) DSA demonstrating the post-traumatic popliteal artery false aneurysm (arrow). (**B**) DSA after endovascular exclusion with a covered stent and restoration of distal perfusion following PTA of the anterior tibial artery (arrows indicate the covered stent and distal runoff). (**C**) Intraoperative view showing extensive purulent inflammation and arterial rupture at the P3/trifurcation level (arrow). (**D**) Intraoperative view after complete debridement and autologous great saphenous vein bypass from the popliteal to the posterior tibial artery (arrow).

We present a rare case of early covered stent infection due to *Salmonella* Enteritidis following urgent endovascular exclusion of a post-traumatic popliteal artery pseudoaneurysm. The novelty is twofold: the unusual occurrence of *Salmonella* graft infection in this peripheral, post-traumatic setting, and a retrospectively identified, culture-confirmed episode of *Salmonella* Enteritidis bacteremia two decades earlier—an association we regard as a hypothesis-generating observation rather than a proven cause. The case underscores the value of meticulous history-taking and of prompt open conversion with radical debridement and autologous reconstruction. The chronological diagnostic, endovascular, surgical and reconstructive stages are shown in Figure 1, and the report follows the CARE guidelines.

## 2. Case Presentation

An 82-year-old man was urgently admitted with acute-on-chronic critical ischemia of the left lower limb. His medical history was significant for radical right nephrectomy for renal cell carcinoma and stage G4 chronic kidney disease. His cardiovascular and metabolic risk profile further included permanent atrial fibrillation, arterial hypertension, type 2 diabetes mellitus, previous coronary artery bypass grafting for ischemic heart disease, and peptic ulcer disease, collectively defining a high operative risk, with a baseline serum creatinine level of 3.46 mg/dL and an estimated glomerular filtration rate (eGFR) of 16 mL/min/1.73 m^2^. Before admission, he was receiving apixaban (2.5 mg twice daily for a cardiovascular indication) together with acetylsalicylic acid (75 mg once daily).

Due to his advanced renal impairment, duplex ultrasonography was selected as the initial imaging modality to minimize exposure to iodinated contrast media [9]. Ultrasound demonstrated a large popliteal artery aneurysmal lesion measuring 47 × 46 mm with an approximate length of 55 mm, initially interpreted as a true degenerative aneurysm. However, given the severity of acute limb ischemia and the need for precise anatomical assessment before revascularization, digital subtraction angiography (DSA) was subsequently performed. DSA revealed a post-traumatic popliteal artery pseudoaneurysm associated with proximal occlusion of the anterior tibial artery (ATA). The ATA was opacified only within the proximal two-fifths of the calf via collateral vessels, whereas the peroneal artery remained patent and only minimal flow was observed within the proximal posterior tibial artery (Figure 1A). The pseudoaneurysm was attributed to a blunt injury sustained approximately three weeks before admission, when the patient had been struck in the left popliteal fossa by a stone fragment.

Considering the patient’s advanced age, substantial comorbidity burden, and high operative risk, an urgent endovascular strategy was adopted. The pseudoaneurysm was successfully excluded using a 6 × 100 mm self-expanding covered stent (Viabahn; W. L. Gore & Associates, Flagstaff, AZ, USA), followed by percutaneous transluminal angioplasty of the occluded anterior tibial artery, which restored satisfactory single-vessel runoff to the foot (Figure 1B). The patient tolerated the procedure well, continued his pre-existing antithrombotic regimen of apixaban and acetylsalicylic acid (clopidogrel-based dual antiplatelet therapy was withheld because of a heightened bleeding risk related to peptic ulcer disease, a platelet count at the lower normal limit of approximately 150 × 10^9^/L, advanced age, and stage G4 chronic kidney disease), and was discharged in stable condition with palpable distal pulses.

Two weeks after the index procedure, the patient was readmitted with progressive pain in the left popliteal fossa, marked swelling, fever reaching 39 °C, and erythematous inflammatory changes around the knee. Laboratory investigations demonstrated pronounced systemic inflammation, including severe leukocytosis (17.12 × 10^9^/L) and a C-reactive protein concentration of 248 mg/L. Repeat duplex ultrasonography showed complete covered stent occlusion, absence of distal arterial flow, and a large hypoechoic perivascular fluid collection highly suggestive of a peri-graft abscess. Given the rapidly deteriorating clinical picture, emergency open surgical exploration was undertaken.

Intraoperatively, extensive purulent inflammation surrounding the covered stent was identified together with a contained arterial rupture at the popliteal trifurcation (Figure 1C). Radical debridement and complete explantation of the infected endograft were performed. Microbiological cultures obtained from both the prosthesis and surrounding tissue yielded *Salmonella* Enteritidis, confirming the diagnosis of early prosthetic vascular graft infection. Blood cultures obtained on readmission were negative, as were stool and urine cultures collected to exclude a persistent enteric or urinary source, indicating a localized graft infection without demonstrable concurrent bacteremia. The isolate was susceptible to amoxicillin-clavulanic acid.

The unexpected isolation of this uncommon vascular pathogen prompted a detailed reassessment of the patient’s previous medical history. Review of historical medical records and regional registry data revealed a remote episode of *Salmonella* Enteritidis bacteremia approximately 20 years earlier. This clinically important information had been unavailable during the index emergency admission because the patient did not recall the illness and no relevant documentation was accessible at the time of the urgent endovascular intervention.

The retrieved records documented that, approximately two decades earlier, the patient had sustained a culture-confirmed episode of *Salmonella* Enteritidis septic shock during the perioperative period of his right radical nephrectomy for renal cell carcinoma. The organism recovered at that time belonged to the same serotype (*S.* Enteritidis) as the pathogen isolated from the infected graft, and no further *Salmonella*-related illness had been documented during the intervening period. The historical infection had been treated initially with amoxicillin-clavulanic acid and subsequently with trimethoprim-sulfamethoxazole, with full clinical recovery, although the exact duration of that treatment was not documented. Molecular strain typing linking the two isolates was not available.

Following extensive irrigation and radical debridement, definitive vascular reconstruction was performed during the same procedure. After thrombectomy of the posterior tibial artery, a popliteal-to-posterior tibial bypass was constructed using the contralateral autologous great saphenous vein (Figure 1D). Based on antimicrobial susceptibility testing, intravenous amoxicillin-clavulanic acid was administered for two weeks, followed by a further two weeks of the same agent orally after discharge (four weeks of targeted therapy in total), under the supervision of the hospital microbiology service. Antibiotics were discontinued after clinical resolution and normalization of inflammatory markers, and the patient subsequently remained under structured clinical and ultrasonographic surveillance.

The postoperative course was uneventful, without wound complications or further deterioration of renal function. At the three-month follow-up, inflammatory markers had normalized and duplex ultrasonography confirmed excellent bypass patency. The patient reported pain-free walking exceeding 1000 m. At the most recent evaluation, two years after surgery, he remained free of recurrent local or systemic infection, with durable graft patency and preserved ambulatory function. At this two-year assessment the vein graft remained patent on duplex ultrasonography with a good flow signal, the ankle-brachial index was greater than 0.6 (consistent with the single-vessel run-off achieved), the debridement wound had healed uneventfully, renal function had not deteriorated (no progression to dialysis), and the patient reported a quality of life appropriate for his age.

Timeline. Blunt popliteal trauma occurred approximately three weeks before presentation. At the index admission (day 0) the patient underwent urgent endovascular covered stent exclusion of the pseudoaneurysm and was subsequently discharged. Two weeks later he was readmitted with an infected, occluded graft and underwent emergency open explantation, radical debridement and popliteal-to-posterior tibial vein bypass. Antibiotic therapy comprised two weeks intravenous followed by two weeks oral. At three months the inflammatory markers had normalized with a patent graft, and at two years he remained free of recurrent infection with a patent graft and preserved ambulation.

## 3. Discussion

The present case highlights several clinically critical aspects of peripheral vascular prosthetic graft infections that extend beyond the rarity of the isolated pathogen. First, it illustrates the extraordinarily aggressive course of *Salmonella* Enteritidis when colonizing endovascular prosthetic material, progressing to total graft occlusion and arterial rupture within two weeks. Second, it emphasizes the complex diagnostic challenges associated with incomplete or unverified historical information during emergency clinical decision-making. Finally, it reinforces the therapeutic principle that radical surgical source control and autologous venous reconstruction remain crucial to achieve long-term infection eradication and limb salvage.

Endovascular repair using covered stents has become an increasingly utilized treatment modality for popliteal artery pathology, offering clear short-term advantages over open surgery in fragile cohorts [2]. Contemporary data have established the technical efficacy of these devices [2,3]. However, prosthetic infection remains one of the most feared complications because it directly threatens both limb viability and patient survival [4]. Fortunately, infections following endovascular popliteal exclusions remain rare, and presentations involving *Salmonella* species are uniquely scarce in the literature [5,8].

*Salmonella* species possess an aggressive tropism for damaged endothelial walls, aneurysmal tissue, and synthetic vascular materials [4,7]. Although most *Salmonella* infections originate as self-limiting gastroenteritis, hematogenous dissemination can occur, particularly in elderly or immunocompromised individuals, leading to persistent bacterial seeding of altered vascular structures [6]. Once synthetic material becomes colonized, the formation of a dense bacterial biofilm severely limits host immune clearance and dramatically impairs the efficacy of systemic antimicrobial therapy, explaining the high failure rates of conservative management [10]. The rapid development of extensive purulent destruction and frank arterial rupture in our patient highlights the need for a high index of clinical suspicion and immediate intervention.

A notable feature of this case is the delayed identification of the remote episode of *Salmonella* bacteremia that occurred two decades prior to presentation. It cannot be definitively proven whether this distant episode resulted in a low-grade, dormant bacterial persistence within the arterial wall or whether it represents an under-recognized systemic vulnerability. However, the biological plausibility of delayed bacterial activation following the introduction of a foreign prosthetic body is well supported in vascular surgery [4]. Consequently, when a historical *Salmonella* infection is known, it may prompt heightened caution and encourage clinicians to consider autologous open repair over synthetic endografting where feasible. This case also illustrates that vital history may be unobtainable in emergency scenarios, underscoring the necessity of strict post-operative surveillance for any patient receiving a covered stent in an urgent setting.

Several non-exclusive mechanisms could theoretically link a remote *Salmonella* episode to late graft colonization. *Salmonella* is a facultative intracellular pathogen capable of surviving within macrophages and other cells of the reticuloendothelial system, and chronic low-level carriage has been described; age-related immunosenescence together with the immune dysfunction associated with stage G4 chronic kidney disease and type 2 diabetes may further impair bacterial clearance, and biofilm formation on prosthetic material can shield persisting organisms from both host defenses and antibiotics. These mechanisms remain speculative in the present case. Equally plausible alternative explanations must be acknowledged, including a recent, undetected transient bacteremia; contamination of the trauma-damaged arterial wall or of the pseudoaneurysm sac before or at the time of stenting; and early perioperative contamination of the endograft. In the absence of molecular typing, none of these pathways can be confirmed or excluded, and the association between the historical bacteremia and the graft infection should be regarded as hypothesis-generating rather than causally established.

The early stent thrombosis observed in this patient is most plausibly explained by the fulminant, culture-positive suppurative process, which was accompanied by peri-graft abscess formation and contained arterial rupture; septic thrombosis of an infected endograft is a well-recognized event. At the same time, the antithrombotic strategy after Viabahn implantation was limited to an anticoagulant (apixaban) combined with a single antiplatelet agent, without the dual antiplatelet therapy often used after covered stent placement. This regimen reflected a deliberate balance against a substantial bleeding risk, but a contribution of suboptimal antiplatelet coverage to stent thrombosis cannot be entirely excluded and is acknowledged as a possible secondary factor.

### 3.1. Contemporary Evidence

Our clinical findings and surgical strategy are highly consistent with contemporary evidence regarding peripheral covered stent infections and vascular graft management. A recent comprehensive multicenter study by Troisi et al. (2025) evaluating the long-term outcomes of Viabahn self-expandable covered stents for popliteal aneurysms demonstrated excellent technical durability and low overall complication rates, which further highlights just how exceptionally rare and unexpected the development of a severe prosthetic infection is in this specific anatomical position [2]. However, when such infectious complications do occur, conservative or localized treatments are uniformly insufficient.

A recent state-of-the-art systematic review by Sfyroeras et al. [8] focusing on stent and stent-graft infections in lower-extremity arteries confirmed that these complications are invariably associated with high rates of major amputation and systemic sepsis unless managed aggressively. Their findings strongly indicate that successful treatment almost always requires complete explantation of the infected prosthetic material combined with radical local debridement, rather than attempting graft preservation with antibiotics alone. This extensive review supports the management strategy adopted in our patient, in whom durable infection control and limb salvage were achieved only after complete removal of the Viabahn graft and radical surgical debridement.

Furthermore, the clinical course described in our report aligns with the experience published by Ascione et al. [5], who emphasized that popliteal endograft infection represents an absolute surgical emergency. Delayed open conversion in their series was directly linked to rapid arterial wall liquefaction, secondary hemorrhage, and a high risk of subsequent major amputation. Their successful management utilized a similar open surgical approach with complete device removal and autologous venous reconstruction. Finally, recent global consensus recommendations by Tabaja et al. regarding vascular graft infections stress that successful management must rely on early microbiological isolation, targeted bactericidal therapy, complete surgical source control, and close multidisciplinary collaboration between vascular surgeons, infectious disease specialists, and microbiologists [10]. The excellent, complication-free two-year outcome observed in our patient is consistent with these contemporary principles. To contextualize our case within the current literature, Table 1 summarizes the main clinical findings of recently published key reports on peripheral stent-graft infections and details their direct relevance to the management of the present case.

Another noteworthy clinical aspect of this case concerns diagnostic imaging in the setting of advanced chronic kidney disease. Duplex ultrasonography was intentionally selected as the first-line screening modality to minimize exposure to iodinated contrast media in a patient with stage G4 CKD [9]. Although the ultrasound successfully identified the aneurysmal lesion, it mischaracterized it as a true degenerative aneurysm, whereas the subsequent digital subtraction angiography correctly identified a post-traumatic pseudoaneurysm. This diagnostic discrepancy underscores the limitations of duplex ultrasound in complex popliteal pathologies and highlights the critical clinical balance between nephroprotection and diagnostic precision. In patients presenting with acute, limb-threatening ischemia, definitive anatomical characterization remains essential for procedural planning, even when contrast administration carries a defined renal risk [9]. Meticulous optimization of contrast volume and aggressive peri-procedural hydration remain important in this vulnerable patient population.

The initial duplex ultrasound misclassified the lesion, whereas digital subtraction angiography established the correct diagnosis. Cross-sectional imaging was not performed because of the patient’s emergent presentation and advanced chronic kidney disease. As a single case, this report cannot establish causality, molecular strain typing was unavailable to confirm persistence of the same *Salmonella* strain, and longer follow-up is needed to confirm long-term durability.

### 3.2. Clinical Lessons

- Preoperative History: A past history of *Salmonella* bacteremia should be actively sought before any planned implantation of prosthetic vascular material, as it may represent a potential risk factor for subsequent device colonization.
- Index of Suspicion: Early post-operative fever, localized popliteal pain, and rapid swelling following covered stent placement should raise suspicion of graft infection rather than being attributed solely to post-implantation inflammatory syndrome.
- Imaging Limitations: While duplex ultrasonography represents an important, contrast-free screening modality, it should not delay definitive diagnostic angiography or computed tomography when clinical suspicion of complex pathology or infection persists.
- Surgical Principles: Radical local soft-tissue debridement, complete explantation of the infected synthetic material, and revascularization utilizing an autologous vein conduit remain crucial for peripheral endograft infections.
- Surveillance: Long-term clinical and ultrasonographic surveillance is strongly recommended following the successful treatment of a mycotic peripheral graft infection to ensure durable patency and detect any late recurrent infectious foci.

Suggested diagnostic–therapeutic pathway for a suspected covered stent infection: (1) recognize early warning signs (post-implantation fever, focal pain, swelling, rising inflammatory markers); (2) obtain blood cultures and duplex ultrasonography, adding contrast-enhanced CT or MR angiography whenever the clinical situation permits; (3) initiate empirical broad-spectrum antibiotics after sampling and involve microbiology/infectious-disease and vascular-surgery teams early; (4) proceed to urgent open exploration with complete graft explantation and radical debridement when infection is confirmed or strongly suspected; (5) restore perfusion with an autologous vein conduit routed through a clean field; and (6) continue culture-directed antibiotics with structured clinical, laboratory, and imaging surveillance.

## 4. Conclusions

Early prosthetic vascular infection caused by *Salmonella* Enteritidis following endovascular covered stent repair of a popliteal artery pseudoaneurysm represents a rare clinical complication. This case suggests that a remote episode of *Salmonella* bacteremia—even when clinically silent for two decades and initially unavailable during an emergency admission— may represent a predisposing factor for subsequent prosthetic colonization. Causality, however, cannot be established from a single observation, and this association should be interpreted as a hypothesis-generating observation: a remote *Salmonella* bacteremia was retrospectively identified, not confirmed as the source of the graft infection. Clinicians should maintain a low threshold for suspecting graft infection and proceeding to prompt surgical conversion in patients presenting with early postoperative inflammatory signs. Early diagnosis, immediate open exploration, complete explantation of all synthetic material, radical soft-tissue debridement, and autologous venous reconstruction remain essential to successful infection eradication, durable limb salvage, and favorable long-term outcomes.

## Figures and Tables

**Table 1:** Clinical implications of recent evidence regarding peripheral stent-graft infection and relevance to the present case.

Reference	Study Design	Main Findings	Relevance to Present Case
Troisi et al. 2025 [2]	Multicenter cohort study	excellent long-term outcomes and patency for Viabahn stents in popliteal repair	underscores the rarity of infectious complications despite high device durability
Ascione et al. 2024 [5]	Clinical case report	popliteal endograft infection successfully treated via open conversion and vein bypass	supports urgent surgical conversion to prevent sepsis and irreversible tissue loss
Sfyroeras et al. 2025 [8]	Systematic literature review	explantation and autologous reconstruction remain the gold standard treatment	is consistent with an aggressive management strategy and underscores the failure of conservative treatment
Tabaja et al. 2024 [10]	State-of-the-art review	emphasizes early diagnosis, targeted antibiotics, source control, and multidisciplinary teams	highlights the need for rapid pathogen identification and prolonged targeted antibiotic regimens
Present Case	Clinical case report	early *Salmonella* Enteritidis infection after Viabahn placement presenting as contained rupture	highlights remote, silent bacteremia as a possible under-recognized risk factor for prosthetic colonization

## Data Availability

The data presented in this study are available on request from the corresponding author. The data are not publicly available due to privacy and ethical restrictions.
